# Supplementary material for: A Global Screen for Assembly State Changes of the Mitotic Proteome by SEC-SWATH-MS
Source: Cell Syst. 2020 Feb 26;10(2):133–155.e6. doi: 10.1016/j.cels.2020.01.001 (PMC7042714; doi:10.1016/j.cels.2020.01.001)

Q9ULG6 | CCPG1_HUMAN | CCPG1 CCP8 CPR8 KIAA1254
Monomer MW [kDa]: 87.34 Monomer expected elution fraction: 41

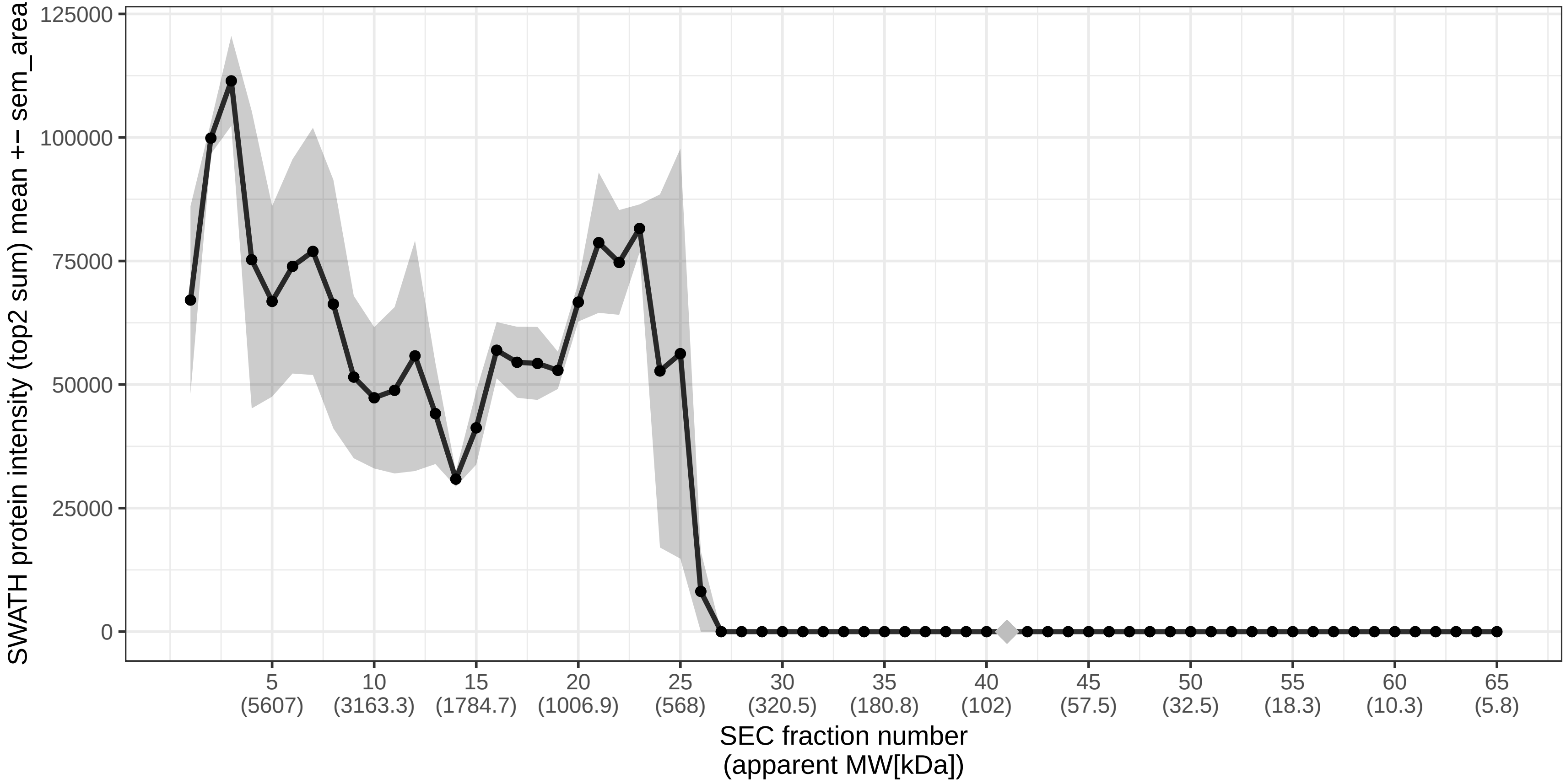

Supplement: Data S1. SEC-SWATH-MS Protein Chromatograms, Related to Figure 1 [file mmc6.zip › SECchrom_Q9ULG6_CCPG1_HUMAN_CCPG1_CCP8_CPR8_KIAA1254.pdf]
